# Enhancing Radiotherapy for Hypoxic Tumors: Integrative Strategies Using Bacteria and Nanoparticles

**DOI:** 10.1049/nbt2/2687439

**Published:** 2025-11-07

**Authors:** Abolfazl Bemidinezhad, Abbas Al-Baghdadi, Anwer Alsarray, Yasaman Abolhassani, Yodgor Kenjayev, Fatemeh Gheybi

**Affiliations:** ^1^Department of Clinical Biochemistry, Faculty of Medicine, Mashhad University of Medical Sciences, Mashhad, Iran; ^2^Department of Pharmacy, College of Pharmacy, University of Kut, Al Kut, Wasit 52001, Iraq; ^3^Department of Medical Biotechnology and Nanotechnology, Faculty of Medicine, Mashhad University of Medical Sciences, Mashhad, Iran; ^4^Department of Basic Medical Sciences, Termez University of Economics and Serviсe, Termez, Uzbekistan; ^5^Nanotechnology Research Center, Pharmaceutical Technology Institute, Mashhad University of Medical Sciences, Mashhad, Iran

**Keywords:** bacterial therapy, hypoxia, nanoparticles, radiosensitization, radiotherapy

## Abstract

Cancer remains a major global health challenge, with radiotherapy (RT) being a cornerstone of treatment. However, the efficacy of RT is significantly hindered by hypoxic tumor microenvironments (TMEs) and nonselective toxicity to healthy tissues. Recent advancements in combining bacteria and nanoparticles have shown promise in addressing these limitations. Cyanobacteria, with their oxygen-producing capabilities, alleviate tumor hypoxia, while anaerobic bacteria selectively target hypoxic regions. Nanoparticles complement these approaches by enhancing bacterial localization and amplifying radiosensitization through reactive oxygen species (ROS) generation and other synergistic therapies. Unlike previous reviews that have mainly focused on either bacterial therapy or nanoparticle-assisted radiosensitization separately, this review provides a comparative and integrative perspective on their combined use, emphasizing the novelty of synergistic strategies. This review explores innovative bacterial–nanoparticle integrations, highlighting their roles in overcoming hypoxia and improving RT outcomes. The potential of these strategies to transform cancer treatment is discussed, alongside challenges and future directions.

## 1. Introduction

Cancer is one of the leading causes of mortality worldwide, with millions of new cases diagnosed annually [1, 2, 3, 4, 5]. The primary treatment strategies for cancer include surgery, chemotherapy, and radiotherapy (RT) [1, 6, 7, 8, 9]. Among these, RT is a cornerstone modality that works by targeting cancerous tissues with ionizing radiation [10, 11, 12, 13, 14]. However, it faces significant limitations, particularly in its ability to achieve selective toxicity and maintain efficacy in hypoxic tumor microenvironments (TMEs) [15, 16].

RT often damages both cancerous and healthy cells indiscriminately, leading to undesirable side effects [12, 17, 18]. Moreover, its therapeutic efficiency is significantly impaired in hypoxic conditions, which are prevalent in many solid tumors [19, 20]. Hypoxia limits the generation of reactive oxygen species (ROS), the primary mediators of DNA damage, and cell death induced by radiation [19, 21, 22]. This phenomenon underscores the importance of radiosensitization strategies that can enhance the tumor's responsiveness to radiation under low-oxygen conditions [13].

Recent research suggests that combining bacteria with nanoparticles could help overcome hypoxia-related resistance in RT [23, 24, 25, 26]. This review provides an integrated perspective on how these approaches may synergize to improve therapeutic outcomes.

While previous reviews have primarily discussed bacterial therapy or nanoparticle-assisted radiosensitization in isolation, the novelty of this review lies in its integrated perspective on how these two approaches can work synergistically. Specifically, we highlight how the complementary roles of cyanobacteria, anaerobic bacteria, and multifunctional nanoparticles can collectively overcome tumor hypoxia, improve radiosensitization, and expand therapeutic potential. By clarifying this scope, the present review aims to fill an important gap in the literature and provide a road map for future investigations.

## 2. Categorization of Bacterial Applications in Radiosensitization

In recent studies, bacteria utilized for radiosensitization have been classified into two main categories: anaerobic bacteria and cyanobacteria [24, 27]. These bacteria employ distinct strategies to address tumor hypoxia, a major barrier to effective RT [27, 28]. Anaerobic bacteria have demonstrated the potential to selectively colonize hypoxic TMEs and facilitate the targeted delivery of nanoparticles, enhancing radiosensitivity in these regions [29]. In contrast, cyanobacteria have been explored for their ability to generate oxygen, mitigating hypoxia and improving the efficacy of RT [30]. In the following subsections, we will discuss these strategies in detail, highlighting the unique roles of specific bacterial species and their associated nanoparticles in overcoming hypoxia and enhancing radiosensitization (Tables 1 and 2).

### 2.1. Anaerobic Bacteria

Anaerobic bacteria have emerged as promising tools in cancer therapy due to their ability to specifically target hypoxic TMEs [25, 31, 34, 35]. This approach has been effectively combined with nanoparticles to address the challenge of hypoxia in RT, enhancing radiosensitivity through targeted delivery and innovative mechanisms (Table 1) [31].

**Figure 1:**
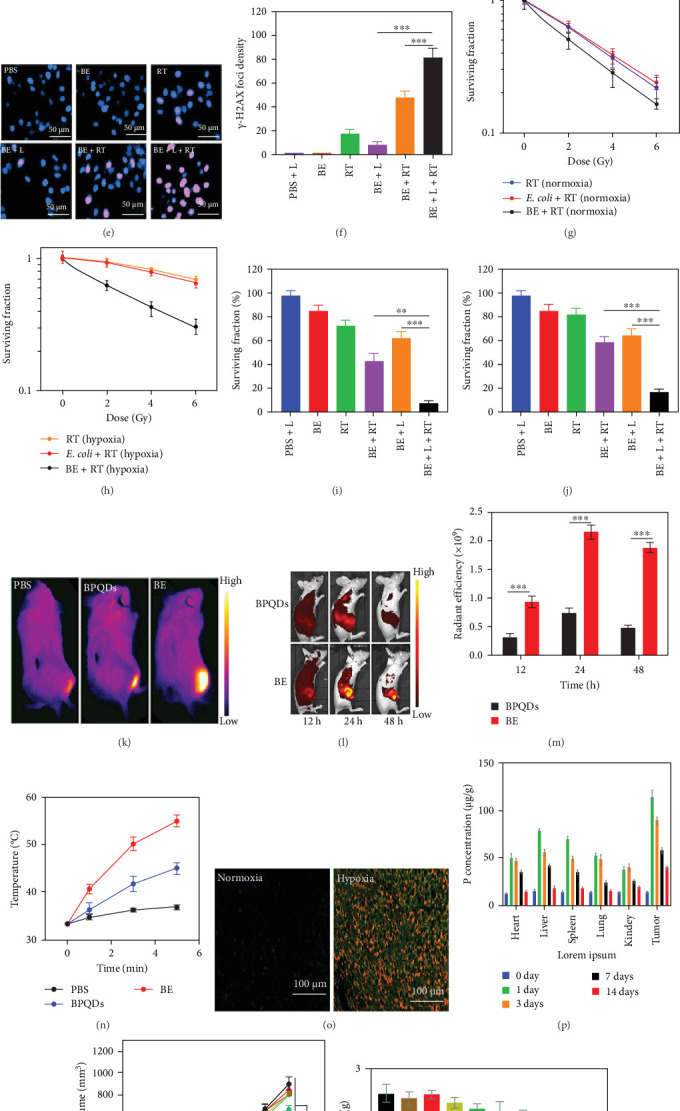
Bacteria-mediated delivery of BPQDs for enhanced photothermal therapy and radiosensitization. (a) TEM images of BPQDs, *E. coli*, and BE hybrids. (b–d) ROS generation under normoxic and hypoxic conditions. (e, f) DNA damage analysis by DAPI and γ-H2AX staining. (g–j) Clonogenic survival and cell viability under various radiation doses and oxygen levels. (k–n) In vivo thermal and fluorescence imaging of BE-treated tumors. (o, p) Tumor hypoxia colocalization and biodistribution. (q–s) Tumor growth inhibition and endpoint tissue images. Statistical significance: *⁣*^*∗*^*p* < 0.05; *⁣*^*∗∗*^*p* < 0.01; *⁣*^*∗∗∗*^*p* < 0.001 (Student's *t*-test). Reproduced with permission from Ji et al. [24], *Journal of Biomaterials Science*, 2023.

Ji et al. [24] conducted a study in which they prepared a hybrid system combining black phosphorus quantum dots (BPQDs) and *Escherichia coli* (*E. coli*) (termed the BE system) using a simple and environmentally friendly liquid exfoliation method. BPQDs are an emerging class of two-dimensional nanomaterials with unique optical and electronic properties, such as excellent photothermal conversion efficiency and high biocompatibility, making them highly suitable for biomedical applications. BPQDs, with an average size of approximately 3 nm and thickness of 1–2 nm, were electrostatically adsorbed onto *E. coli*, approximately 2.3 μm in size. This modification resulted in a loading efficiency of 68.62%, as confirmed by UV–Vis spectra, without affecting the bacterial size or viability under dark conditions. However, BPQDs demonstrated strong photothermal properties, significantly reducing bacterial viability under 808 nm laser irradiation. BE system exhibited robust absorption in the near-infrared (NIR) range and rapid heat generation, enabling efficient photothermal therapy (PTT). Cellular uptake studies in CT26 cells revealed high internalization rates under both normoxic and hypoxic conditions. The system also demonstrated excellent biocompatibility and negligible hemolytic activity. Under hypoxic conditions, BE significantly enhanced ROS production upon X-ray (2 Gy) and laser (808 nm) irradiation, leading to a 4.56-fold increase in γ-H2AX foci, indicative of substantial DNA damage. This innovative integration of BPQDs with bacteria highlights a promising strategy for oxygen-independent tumor therapies, addressing challenges like rapid oxygen leakage and low efficacy in hypoxic microenvironments (Figure 1) [24].

**Figure 2:**
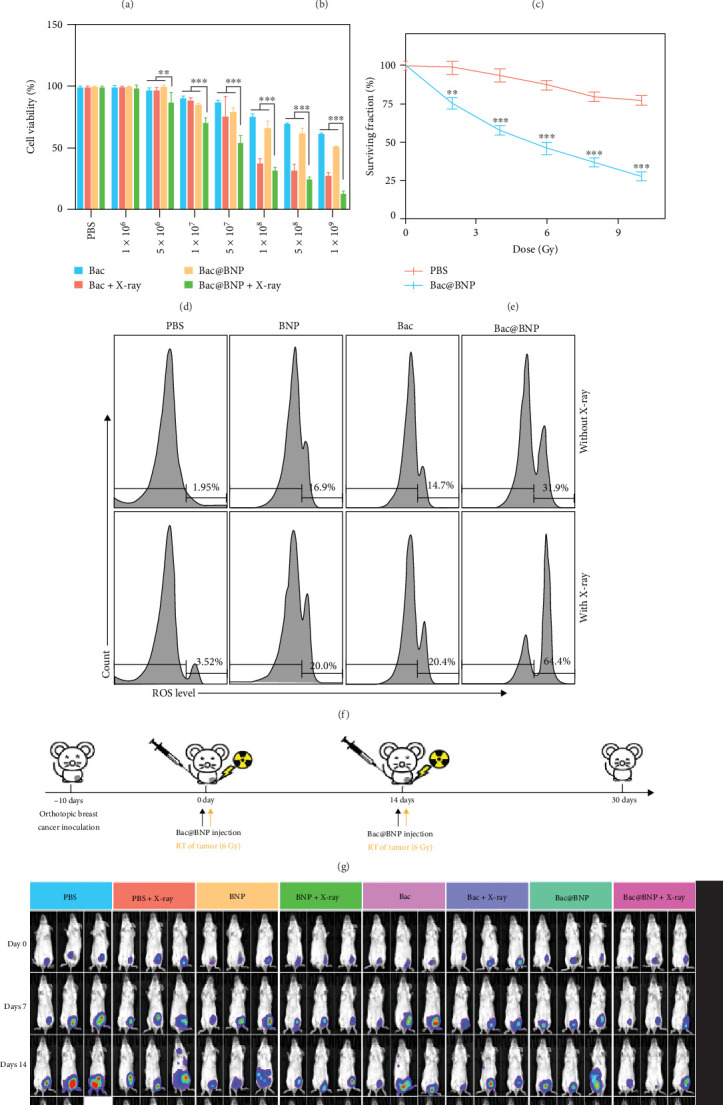
Engineered bacteria enhance radiotherapy efficacy against breast carcinoma. (a) Western blot confirming ClyA expression in *Bac* with L-arabinose induction. (b, c) Cell cycle analysis of 4T1 cells under different Bac treatments. (d, e) Cell viability and clonogenic survival with or without X-ray irradiation. (f) ROS production assessed by DHE staining and flow cytometry. (g) Schematic of Bac@BNP therapeutic strategy. (h) In vivo bioluminescence imaging of 4T1-luc tumor-bearing mice under different treatment conditions. Statistical significance: *⁣*^*∗*^*p* < 0.01; *⁣*^*∗∗*^*p* < 0.005; *⁣*^*∗∗∗*^*p* < 0.001 (two-tailed Student's *t*-test, GraphPad Prism 8). Reproduced with permission from Pan et al. [29] Copyright 2022, *American Chemical Society*.

Pan et al. [29] developed a novel nanosystem, Bac@Bi_2_S_3_ nanoparticle (BNP), by engineering *E. coli* (eBac) to overexpress cytolysin A (ClyA), a pore-forming protein, for enhanced radiosensitization in tumor cells. The pBAD18-ClyA plasmid facilitated the extracellular secretion of ClyA under L-arabinose induction, as confirmed by western blot, SDS-PAGE, and qPCR analyses. This protein successfully shifted the tumor cell cycle from the radioresistant S phase (36.89%) to the radiosensitive G2/M phase (45.23%), increasing the efficacy of RT. BNPs, a high-*Z* material with superior X-ray absorption properties, were attached to eBac via an amide condensation reaction to form the Bac@BNP nanosystem. BNPs demonstrated remarkable stability, with minimal changes in zeta potential (approximately −39.4 mV) and size distribution over 14 days [29]. Upon tumor colonization, BNPs were selectively released in response to matrix metalloproteinase-2 (MMP-2) activity, ensuring targeted delivery. Under X-ray irradiation, Bac@BNP generated ROS via the photoelectric effect, amplifying DNA damage and reducing hypoxia. In vitro assays revealed significantly lower survival fractions and colony formation in 4T1 breast cancer cells treated with Bac@BNP and X-rays compared to controls. Clonogenic and fluorescence assays confirmed synergistic effects of ClyA and BNPs, mediated by ROS and DNA damage. This dual-functional nanosystem demonstrated exceptional tumor suppression and potential for precise, minimally invasive RT (Figure 2) [29].

Wang et al. [31] developed the SO@Hf–MOF–Pt biohybrid by integrating *Shewanella oneidensis* MR-1 (SO) with hafnium-based metal-organic frameworks functionalized with platinum nanoparticles (Hf–MOF–Pt). The Hf–MOF nanoparticles, synthesized with an average size of 100 nm, were modified with Pt nanoparticles (average size ~150 nm), as confirmed by SEM, TEM, PXRD, and XPS analyses. Electrostatic interactions facilitated the attachment of these nanoparticles to SO, yielding biohybrids with favorable stability and preserved bacterial activity. This hybrid system exhibited enhanced lactate metabolism, consuming excess lactate in the TME and catalyzing hydrogen peroxide (H_2_O_2_) into oxygen. This dual mechanism alleviated hypoxia and downregulated hypoxia-inducible factor-1α (HIF-1α), reshaping the TME [31]. Under X-ray irradiation, the biohybrid synergized metabolic and radiosensitization effects, generating ROS and inducing significant DNA damage in CT26 colorectal cancer cells. The presence of high-*Z* hafnium increased X-ray absorption, while Pt nanoparticles catalyzed oxygen production, mitigating hypoxia. This resulted in superior therapeutic efficacy, with SO@Hf–MOF–Pt + X-ray showing the highest ROS production, DNA damage, and cytotoxicity compared to controls. Importantly, the biohybrid exhibited minimal toxicity toward normal cells, highlighting its biocompatibility. These findings underscore the potential of SO@Hf–MOF–Pt as an innovative radiosensitizer, combining metabolic regulation with enhanced RT for colorectal cancer treatment [31].

**Figure 3:**
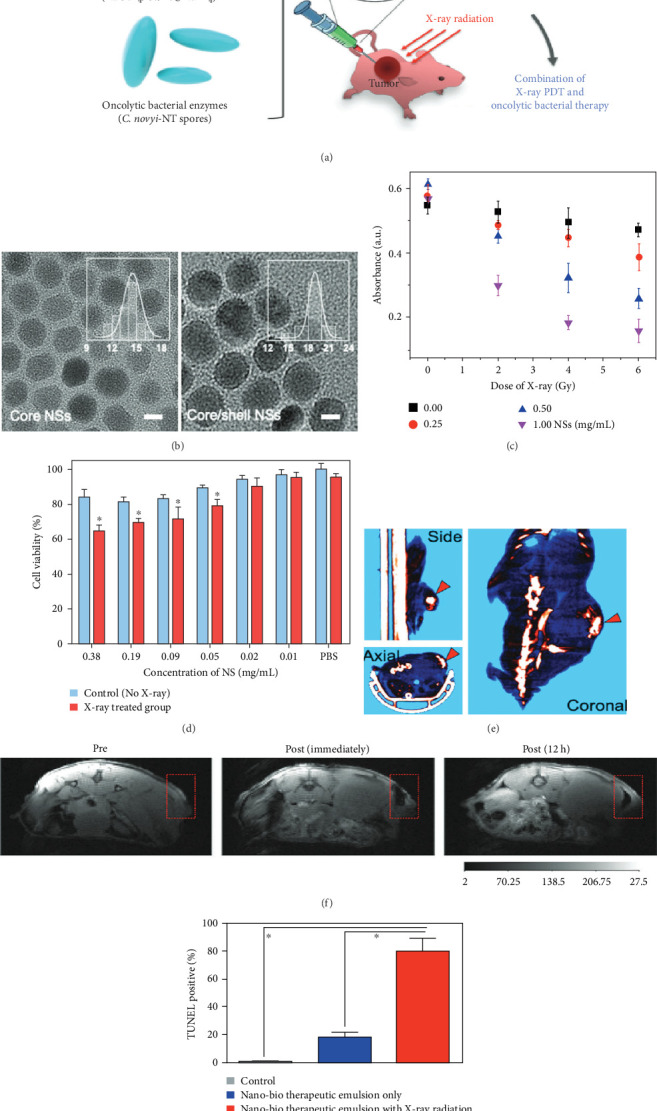
Tumor-targeting nano-bio emulsions enable synergistic X-ray photodynamic and bacterial therapy. (a) Schematic of the therapeutic emulsion (nanoscintillators, *C. novyi*-NT spores, and ethiodized oil) for combined PDT and bacterial treatment. (b) TEM images of core and core–shell nanoscintillators. (c) ROS generation by RB-loaded nanoscintillators under irradiation. (d) Viability of PC3 cells after treatment with RB-NSs and X-ray. (e, f) Micro-CT and MR imaging demonstrating tumor targeting. (g) TUNEL analysis of apoptosis and DNA damage in tumor tissues. Statistical significance: ∗*p* < 0.05 (Student's unpaired *t*-test, GraphPad Prism). Reproduced with permission from Park et al. [32], *Advanced Healthcare Materials*, Copyright 2022, Wiley–VCH.

Park et al. [32] explored a novel combination therapy utilizing *Clostridium novyi*-NT spores and NaGdF_4_–based core–shell nanoparticles (NSs) for CT-image-guided cancer treatment. *Clostridium novyi*-NT spores selectively germinate in hypoxic tumor regions, secreting enzymes to lyse cancer cells, while normoxic peripheral cells often remain viable, leading to recurrence. To address this, the research introduced a nano-bioemulsion integrating PS-coated lanthanide-doped NSs and *C. novyi*-NT spores, emulsified with iodinated oil (Lipiodol) for intratumoral injection. The nanoparticles emitted fluorescence via FRET upon X-ray exposure, producing ROS for photodynamic therapy (PDT). These nanoparticles were also MRI-visible, enabling precise monitoring [32]. The therapeutic efficacy was evaluated in a prostate tumor-bearing mouse model, revealing enhanced apoptosis in both hypoxic and normoxic tumor regions. The NSs exhibited a spherical structure (19.0 ± 1.8 nm), significant ROS production, and superior MRI contrast (*r*_2_/*r*_1_ = 6.68). Immunohistochemistry confirmed the treatment's success in addressing tumor heterogeneity. This integrated PDT and bacteriolytic approach overcame limitations of conventional PDT, such as poor light penetration (<5 cm in tissue) and inadequate PS distribution, offering a significant advancement in targeting spatially heterogeneous tumors with precision imaging and therapy (Figure 3) [32].

Collectively, these studies underscore the potential of anaerobic bacteria paired with nanoparticles in overcoming hypoxia and enhancing radiosensitivity. While each approach demonstrates innovative strategies, the challenges of scalability, long-term biosafety, and precise delivery warrant further exploration to translate these promising platforms into clinical applications.

### 2.2. Cyanobacteria

The use of cyanobacteria in combination with nanoparticles represents a promising approach to addressing tumor hypoxia and enhancing radiosensitization in cancer therapy [23, 36]. Cyanobacteria, due to their photosynthetic oxygen production capability, offer an innovative solution to alleviate hypoxia in the TME [23]. Meanwhile, nanoparticles provide complementary functionalities, such as ROS generation, RT sensitization, and PDT [23, 37].  Section 2.2 evaluates four studies that explore the synergistic applications of cyanobacteria and nanoparticles, with an emphasis on their novelties, limitations, and therapeutic potential (Table 2) [23].

**Figure 4:**
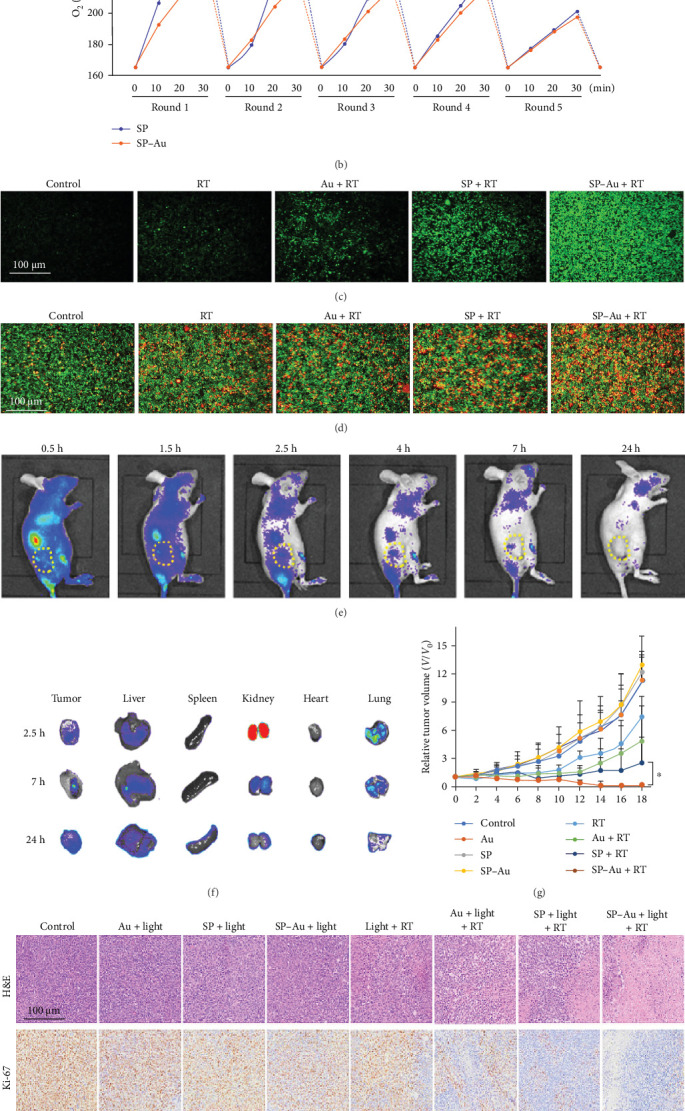
Photosynthetic bacteria-based biohybrids enhance radiotherapy via oxygen generation and photosynthetic activity. (a) Schematic of SP–Au mechanism for ROS generation and glutathione oxidation. (b) Oxygen concentration profiles of SP and SP–Au under light irradiation. (c, d) Fluorescence imaging of ROS production and cell viability in 4T1 cells. (e, f) In vivo and ex vivo fluorescence imaging showing biodistribution of SP–Au. (g) Tumor growth curves in treated mice. (h) Histological staining (H&E, CD31, Ki-67, and HIF-1α) demonstrating effects on angiogenesis, proliferation, and hypoxia. Statistical significance: ∗*p* < 0.05; (Student's *t*-test). All data are presented as mean ± standard deviation or mean. Reproduced from Hua et al. [33] *Journal of Nanobiotechnology*, 2024, licensed under CC BY 4.0.

Hua et al. [33] introduced a biohybrid system combining Spirulina platensis and gold (SP–Au) nanoclusters to enhance RT through photosynthetic oxygen production and catalytic activity. The SP–Au biohybrid efficiently generates ROS, particularly superoxide anions (O_2_^•−^), through cascade photocatalysis. Under red light illumination, oxygen production in SP–Au increased by 1.5 times within 30 min and remained stable over at least five cycles, demonstrating its photostability. SP–Au also showed enhanced ROS production, outperforming Au nanoclusters alone due to supplementary oxygen generation by SP. Cellular studies revealed significant alleviation of hypoxia and ROS accumulation when SP–Au was combined with RT and light, reducing tumor cell survival to just 19% at a 6-Gy radiation dose under hypoxic conditions. In vivo, SP–Au exhibited targeted tumor accumulation, with fluorescence signals detected in tumors starting 2.5 h postinjection, persisting for up to 24 h. Degradation studies showed SP–Au was metabolized into smaller fragments and excreted within this timeframe. Tumor models treated with SP–Au, light, and RT demonstrated superior therapeutic outcomes, including significant tumor necrosis and reduced markers of angiogenesis and proliferation (CD31+ and Ki-67+ cells). However, its dependence on red light limits efficacy in deeper tissues, highlighting a need for improved light penetration strategies (Figure 4) [33].

Jiang et al. [27] developed photosynthetic microcapsules (PMCs) encapsulating *Synechococcus elongatus* and upconversion nanoparticles (UCNPs) to combat melanoma resistance through lipid peroxidation and ferroptosis. Cyanobacteria were selected for their oxygen production capacity, with *Synechococcus elongatus* evolving under physiological conditions (37°C, 10% serum) to achieve robust growth. UCNPs, synthesized from NaErF_4_:Tm@NaYF_4_ with a size of 20 nm, were used to convert tissue-penetrating NIR-II radiation (1550 nm) into red light (630–680 nm), matching chlorophyll-α absorption. This energy source enabled efficient photosynthesis in PMCs, achieving a dissolved oxygen level of 7.3 mg/L after 40 min of exposure to NIR-II radiation. Optimal oxygen generation was observed with PMCs containing 0.7 µg UCNPs and 1 × 10^6^*Synechococcus elongatus* cells per microcapsule, sustaining >85% photosynthetic activity over 7 days in RPMI 1640 media [27]. In melanoma cells (B16), PMCs improved hypoxic conditions, reducing hypoxia-associated fluorescence by 90%. The synergy of PMCs and X-ray radiation (6 Gy) induced lipid peroxidation (fourfold higher than LOX-mediated reactions) and activated ferroptosis by suppressing glutathione peroxidase 4 and depleting glutathione. Tumor metastasis was significantly inhibited, and survival rates in melanoma-bearing mice improved. However, challenges remain, including the potential leakage of cyanobacteria and ensuring stability under physiological conditions. The microcapsule's design, incorporating polylysine coating, mitigates cyanobacteria leakage, but requires further refinement for clinical translation [27].

**Figure 5:**
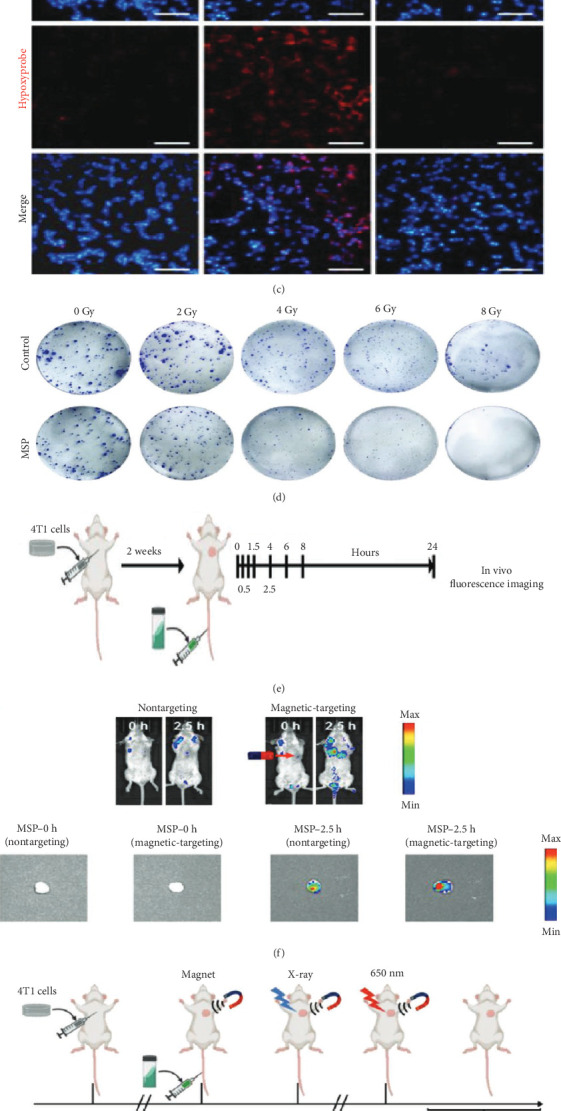
Photosynthetic biohybrid nanoswimmers mitigate hypoxia and enable multimodal imaging for guided radio-photodynamic therapy. (a) Schematic of magnetic *S. platensis*-based nanoswimmers for targeted delivery and combined PDT/RT. (b) Oxygen concentration curves under light irradiation for water, SP, and MSP-2.5. (c) Fluorescence imaging of 4T1 cells stained for nuclei and hypoxia. (d) Colony formation assay of 4T1 cells after treatment. (e, f) In vivo biodistribution and tumor accumulation of MSP with/without magnetic guidance. (g) Schematic of combined PDT and RT strategy in orthotopic 4T1 tumors. (h) Histological staining (H&E, CD31, Ki-67, and HIF-1α) showing effects on proliferation, hypoxia, and vascularization. Reproduced with permission from Zhong et al. [30], *Advanced Functional Materials*, Copyright 2022, WILEY–VCH VERLAG GMBH & CO. KGAA.

Zhong et al. [30] developed magnetic SP (MSP) biohybrid nanoswimmers functionalized with Fe_3_O_4_ nanoparticles for tumor-targeted imaging and therapy. These magnetic nanoswimmers exhibit a unique helical morphology and intrinsic chlorophyll fluorescence, allowing for light-driven oxygen production to mitigate hypoxia in TMEs. Through chlorophyll-mediated ROS generation, MSP amplifies the efficacy of RT and PDT. The Fe_3_O_4_ nanoparticles, approximately 10 nm in size, ensure high superparamagnetic properties, enabling precise external magnetic actuation. Using a permanent magnet, the MSP can be directed toward tumor sites, as evidenced by controlled movement observed through microscopy and dynamic tests. This feature enhances tumor-specific targeting, allowing for more accurate treatment delivery [30]. Furthermore, MSP's photosynthetic oxygen generation capability was validated through increased oxygen concentrations under light exposure, significantly alleviating hypoxia in vitro. Remarkably, in hypoxic cancer cells treated with MSP and irradiated with X-rays, survival fractions decreased by over 90% at 8 Gy compared to RT alone. Despite these therapeutic advancements, challenges remain. The complex fabrication process and potential toxicity of Fe_3_O_4_ nanoparticles at higher doses necessitate comprehensive investigations into safety, biocompatibility, and scalability. Nevertheless, the synergistic integration of external magnetic guidance and multimodal therapeutic functions underscores the transformative potential of MSP in cancer therapy (Figure 5) [30].

**Figure 6:**
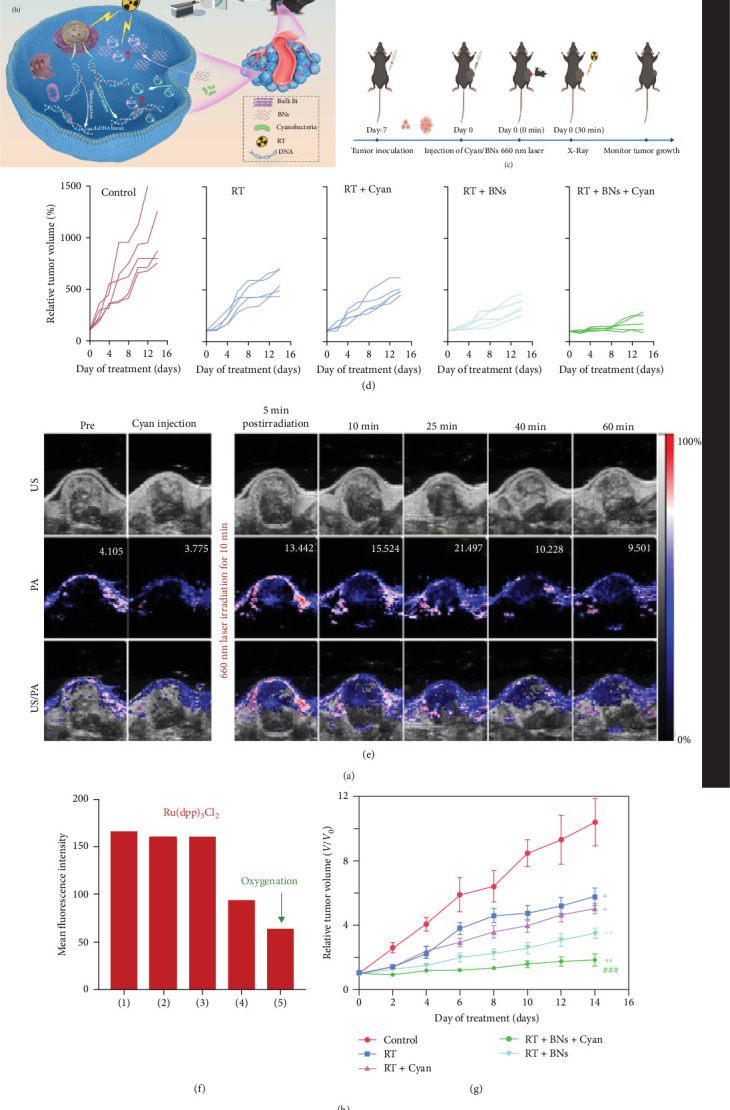
Photosynthetic cyanobacteria generate oxygen to support 2D bismuthene-enhanced radiotherapy. (a) Preparation of 2D bismuthene nanosheets by freeze–thaw and sonication. (b, c) Schematic of cyanobacteria/BN-based radiotherapy platform for hypoxia alleviation and ROS generation. (d) Tumor growth curves in LLC-bearing mice under different treatment groups. (e) US and PA imaging of tumor oxygenation after cyanobacteria administration and laser irradiation. (f) In vitro oxygen production and cellular effects under laser stimulation. (g) Tumor volume changes demonstrating enhanced efficacy of combined cyanobacteria and BN treatment. Statistical significance: n.s., not significant; *⁣*^*∗*^*p* < 0.05, significant; *⁣*^*∗∗*^*p* < 0.01, moderately significant; and *⁣*^*∗∗∗*^*p* < 0.001, highly statistically significant compared to the control group. ^###^*p* < 0.001 compared to the RT group (GraphPad Prism 8). Reproduced from Chai et al. [23], *Bioactive Materials*, 2022, licensed under CC BY 4.0.

Chai et al. [23] integrated *Synechococcus elongatus* PCC7942 cyanobacteria with two-dimensional bismuthene nanosheets, creating a promising biomimetic platform for overcoming RT resistance in hypoxic tumors. The cyanobacteria, cultured in mid-exponential phase for optimal photosynthetic activity, generate oxygen through photosynthesis, significantly alleviating tumor hypoxia upon 660 nm laser irradiation. Their thylakoid membranes, organized into concentric shells, house photosynthetic pigments, and electron transport chains, enabling robust oxygen evolution and suppression of HIF-1α expression. Complementing this, the nanosheets, prepared through freeze–thaw cycles and sonication, provide a high atomic number (*Z*) radiosensitization capability. With an average size of 200 nm, they remain stable under physiological pH conditions and exhibit efficient cellular uptake. In murine lung (LLC) and breast (4T1) cancer models, the combined system enhances ROS generation, boosts RT efficacy, and induces significant tumor apoptosis without inducing photothermal effects. However, batch-to-batch variations in nanosheet functionalization warrant further optimization (Figure 6) [23].

These studies collectively demonstrate the transformative potential of cyanobacteria–nanoparticle biohybrids in overcoming tumor hypoxia and enhancing RT. However, challenges such as scalability, stability, and safety must be addressed to translate these innovative systems into clinical practice.

## 3. Mechanisms of Nanoparticle Interaction With Bacteria

**Figure 7:**
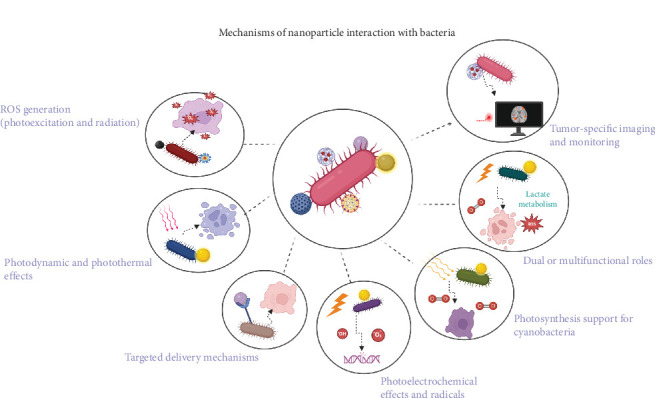
Nanoparticle–bacteria interactions in tumor therapy. Nanoparticles enhance bacterial and tumor cell killing via ROS, photothermal/photodynamic effects, and targeted delivery. They support radical generation, cyanobacterial photosynthesis for oxygenation, multifunctional therapy (lactate metabolism, O_2_, and ROS), and tumor-specific imaging (MRI/fluorescence).

Nanoparticles provide multiple mechanisms that complement bacterial therapy in radiosensitization. The following subsections highlight these mechanisms in detail (Figure 7) [38, 39, 40].

### 3.1. ROS Generation Through Photoexcitation and Radiation

Nanoparticles, particularly high-*Z* materials and quantum dots, act as catalysts to amplify ROS production under irradiation [24, 31, 41, 42]. For example, BPQDs attached to *E. coli* generate ROS upon exposure to 808 nm laser light and X-rays. This phenomenon directly reflects the findings in Section 2.1, where the BE system (*E. coli*–BPQDs) demonstrated robust ROS generation under hypoxic conditions, amplifying radiosensitization [24]. Similarly, Hf–MOFs functionalized with platinum amplify ROS production via the photoelectric effect, catalyzing oxygen release to alleviate hypoxia. This approach effectively addresses two critical barriers in RT: oxidative stress induction and hypoxia reduction. However, one challenge is the potential cytotoxicity of nanoparticles to healthy cells, which requires precise dosage optimization. Future research should focus on fine-tuning the nanoparticle concentration and improving targeting specificity to minimize side effects [24, 31].

### 3.2. Photodynamic and Photothermal Effects

PDT and PTT mechanisms are enhanced when nanoparticles are combined with bacteria [37, 43]. In PDT, nanoparticles like Au nanoclusters or UCNPs absorb light and transfer energy to nearby molecules, producing ROS [27, 33]. For instance, Spirulina–Au nanocluster hybrids enhance PDT by supplying oxygen for ROS generation, which is critical under hypoxic conditions. As described in Section 2.2, the SP–Au system showed this dual role, where photosynthetic oxygen production by Spirulina significantly enhanced PDT efficacy [33]. In PTT, nanoparticles like BPQDs convert absorbed light into heat, directly inducing cancer cell apoptosis. PDT and PTT are complementary mechanisms, with PDT focusing on ROS-mediated cytotoxicity and PTT utilizing thermal damage. While both are promising, the integration of bacteria offers an edge in addressing hypoxia, a major limitation of conventional phototherapies. However, ensuring light penetration in deeper tissues remains a technical challenge. The development of nanoparticles with higher efficiency in NIR light absorption may enhance clinical applications [27, 33].

### 3.3. Targeted Delivery Mechanisms

Active targeting is achieved through innovative strategies such as magnetic guidance or enzyme-sensitive release [44]. Iron oxide nanoparticles conjugated to cyanobacteria allow magnetic navigation to hypoxic tumor regions, ensuring precise delivery [30]. This mirrors the MSP nanoswimmers discussed in Section 2.2, which utilized Fe_3_O_4_ nanoparticles for magnetically guided tumor targeting [30]. Enzyme-responsive systems, such as *E. coli* with MMP-sensitive coatings, release nanoparticles selectively within the TME, minimizing off-target effects [29]. While magnetic and enzyme-responsive targeting show great promise, their scalability and reproducibility in clinical settings require further investigation. Additionally, regulatory challenges regarding the use of genetically modified bacteria must be addressed to pave the way for clinical translation. Establishing standardized protocols for bacterial modification and nanoparticle synthesis could significantly accelerate progress [29, 30].

### 3.4. Photoelectrochemical Effects and Radicals

Certain nanoparticles induce photoelectrochemical reactions under irradiation, creating a cascade of ionic and radical formations [45, 46]. For instance, hafnium nanoparticles generate hydroxyl radicals (•OH) via water splitting under X-rays, augmenting DNA damage [31]. A similar principle was observed in Section 2.1, where the SO@Hf–MOF–Pt biohybrid leveraged hafnium's high-*Z* properties to boost radical generation under irradiation [31]. Similarly, NaGdF_4_–based core–shell nanoparticles emit fluorescence and produce radicals when excited, complementing bacterial activity in hypoxic tumors [32]. These photoelectrochemical effects demonstrate the versatility of nanoparticles in amplifying RT outcomes. Comparing this approach to ROS generation, one clear advantage is the ability to produce radicals directly from water molecules, leveraging the tumor's aqueous microenvironment. However, further studies are needed to quantify and control radical production to avoid unintended damage to adjacent healthy tissues [31, 32].

### 3.5. Photosynthesis Support for Cyanobacteria

Nanoparticles facilitate photosynthesis in cyanobacteria by acting as light-harvesting or energy-transducing agents [47]. For instance, UCNPs absorb NIR light and emit it as visible light, enabling deeper tissue penetration for cyanobacterial oxygen production [27]. This directly corresponds to the PMCs with *Synechococcus elongatus* in Section 2.2, where UCNPs enabled sustained oxygen production under NIR-II irradiation [27]. This enhances the oxygenation of the TME, countering hypoxia and improving radiosensitization [48, 49]. While this approach is innovative, the reliance on photosynthesis limits its applicability to tumors with sufficient light penetration [50]. Combining photosynthetic systems with nanoparticles capable of deeper light penetration or alternative oxygen-generation strategies, such as chemical catalysis, could broaden its utility [51, 52].

### 3.6. Dual or Multifunctional Roles

Multifunctional nanoparticle systems combine several mechanisms [53]. For example, the SO@Hf-MOF-Pt biohybrid integrates Shewanella with hafnium and platinum, enabling simultaneous lactate metabolism, oxygen generation, and ROS production under X-rays [31]. As highlighted in Section 2.1, this multifunctional system not only alleviated hypoxia but also induced significant DNA damage in CT26 tumor cells. These systems not only alleviate hypoxia but also synergize metabolic regulation with RT for superior outcomes [54, 55]. Multifunctional systems, while highly effective, are often complex and challenging to produce on a large scale. Simplifying these systems without compromising their efficiency could make them more feasible for clinical applications. Collaboration between biologists, chemists, and engineers is crucial for achieving this goal [31].

### 3.7. Tumor-Specific Imaging and Monitoring

Lanthanide-doped nanoparticles integrated with bacteria provide real-time imaging capabilities through MRI or fluorescence [56, 57]. This was exemplified in Section 2.1 by the *Clostridium novyi*-NT spores combined with NaGdF_4_ nanoparticles, which allowed both therapeutic ROS generation and MRI-guided tumor monitoring [32]. The integration of imaging and therapeutic capabilities is a significant advancement, offering a theranostic approach. However, the potential immunogenicity of bacterial carriers remains a concern. Developing biocompatible coatings or using less immunogenic bacterial strains could mitigate this issue [32].

The interaction of nanoparticles with bacterial systems offers a groundbreaking approach to enhancing RT through improved ROS production, targeted delivery, and tumor-specific imaging [58]. Despite the promising results, challenges such as scalability, immunogenicity, and light penetration must be addressed to translate these systems into clinical practice [58, 59]. Future research should prioritize multidisciplinary collaborations to refine these technologies and ensure their safe and effective application in oncology [59].

## 4. Challenges, Limitations, and Future Perspective

While anaerobic bacteria exploit hypoxic niches and cyanobacteria generate oxygen, both strategies face critical biological and technical barriers that limit clinical translation. Key issues include biosafety, immune clearance, and effective light or nutrient delivery in deep tumor tissues [23, 24].

Another important translational challenge concerns the experimental conditions used in several reported studies. Many of these rely on relatively high radiation doses or strong light intensities (e.g., lasers at specific wavelengths) that may not be clinically feasible due to risks of collateral tissue damage or technical limitations in patient settings [23, 29]. While such parameters are useful for proof-of-concept demonstrations, they raise questions about clinical relevance. Future work should, therefore, focus on optimizing nanoparticle–bacteria systems to achieve therapeutic efficacy under clinically acceptable radiation doses and light intensities, or alternatively, on developing sensitizers and delivery strategies that can lower the required energy thresholds [23, 29].

Biosafety remains one of the foremost obstacles [31, 60, 61, 62, 63]. Many anaerobic bacterial strains, such as *Clostridium* or *Salmonella*, are derived from opportunistic pathogens [30, 32]. Even when attenuated, the risk of systemic infection or sepsis cannot be fully excluded, necessitating rigorous genetic modifications and safety switches. Cyanobacteria are generally regarded as safer, but their long-term effects in mammalian systems, particularly regarding potential microbe–host interactions, remain insufficiently understood [30, 32].

Immune clearance also represents a significant barrier [64, 65, 66]. Systemically administered bacteria are rapidly detected and eliminated by the host immune system, reducing therapeutic efficacy. Strategies such as surface functionalization with polymers, encapsulation in nanoparticles, or using less immunogenic bacterial strains may extend circulation time, but balancing immune evasion with therapeutic functionality remains a challenge [27, 31].

Light penetration constraints particularly limit cyanobacteria-based strategies. Photosynthetic oxygen production depends on sufficient illumination, but visible light does not penetrate deeply into tissues. NIR light offers greater penetration, yet its efficiency is still inadequate for large or deep-seated tumors. Emerging approaches, such as integrating UCNPs that convert NIR to visible light, or implantable micro-light delivery devices, may help overcome this barrier, but they add technical complexity and regulatory hurdles [27, 30].

In addition to biological and technical hurdles, economic feasibility is another critical factor for clinical translation. The production of engineered bacteria and nanoparticle hybrids often involves sophisticated biotechnological processes and costly nanomaterials, raising concerns about large-scale manufacturing and affordability [23, 33]. While preclinical studies typically focus on proof-of-concept efficacy, future work must also evaluate cost-effectiveness compared with existing standards of care, such as RT combined with conventional radiosensitizers. Addressing economic sustainability early in development will be essential to ensure that these therapies are not only clinically effective but also accessible to patients in real-world healthcare systems [27, 31].

From a translational perspective, anaerobic bacteria offer the clear advantage of naturally and selectively colonizing hypoxic tumor cores, making them highly suitable for targeting regions that are typically resistant to RT [29, 31]. However, their dependence on hypoxia restricts their therapeutic effects to specific tumor zones, leaving normoxic regions less affected [31]. In contrast, cyanobacteria provide systemic benefits by actively generating oxygen through photosynthesis, thereby alleviating tumor hypoxia across both hypoxic and normoxic regions. Yet, this advantage comes with the drawback of requiring external light penetration, which is significantly limited in deep-seated or poorly accessible tumors [30, 33].

Despite these challenges, bacteria hold tremendous potential for development as therapeutic tools due to their inherent biocompatibility with the human body [67]. Bacteria naturally thrive in hypoxic tumor environments, a trait that can be leveraged for precise tumor targeting [67, 68]. Anaerobic bacteria may be more immediately translatable to clinical use, since they can function without external triggers such as light, but they require careful genetic attenuation to avoid pathogenicity [31]. Cyanobacteria, on the other hand, are generally considered safer, but demand advanced optical or nanomaterial-based delivery systems to overcome the depth limitations of light. Thus, each approach carries distinct advantages and disadvantages that must be weighed for clinical translation [27].

One of the most promising future directions is the use of genetic engineering to modify bacteria [69, 70]. These modifications could enhance their ability to release oxygen, produce radiosensitizing molecules, and reduce pathogenicity [69, 71]. For instance, genetically engineered bacteria capable of specifically producing molecules that boost immune responses could significantly enhance the efficacy of RT [69]. Combining these approaches with existing therapies, such as immunotherapy, could also improve treatment outcomes [69, 72, 73, 74, 75]. For example, designing bacteria that simultaneously stimulate the immune system and improve tumor oxygenation presents an innovative and synergistic strategy [68, 72]. With the advancement of cutting-edge technologies, including precise release systems and nanocarriers, the clinical applications of these bacteria could be significantly expanded [42, 76, 77]. Due to their natural abilities to penetrate tumors and function effectively in hypoxic environments, these biological agents offer a potentially safer and more biocompatible alternative to many synthetic chemical agents [68]. Ultimately, integrating interdisciplinary knowledge from microbiology, bioengineering, and nanotechnology offers a promising pathway to overcome current limitations and broaden the therapeutic applications of this innovative approach [78, 79, 80]. Such advancements could pave the way for the widespread clinical adoption of bacterial therapies, ushering in a new era in cancer treatment [78].

## 5. Conclusion

This review underscores the synergistic potential of bacteria–nanoparticle systems in radiosensitization. While notable progress has been achieved in preclinical models, future efforts must focus on biosafety, delivery precision, and clinical scalability to realize their full therapeutic promise. The integration of anaerobic bacteria, cyanobacteria, and multifunctional nanoparticles provides complementary advantages: selective tumor colonization, oxygen generation, ROS amplification, and imaging-guided monitoring. Together, these features address the long-standing challenge of tumor hypoxia, one of the major barriers in RT.

However, several key research gaps remain. First, biosafety and immunogenicity represent the most urgent priorities, as ensuring patient safety is essential before any clinical translation. Second, immune clearance mechanisms and strategies to prolong bacterial persistence without compromising safety require systematic investigation. Third, light penetration constraints—particularly for cyanobacteria-based approaches—demand innovative solutions, such as NIR-responsive or upconversion nanomaterials. Finally, large-scale production and reproducibility of genetically engineered bacteria and complex bio-nano hybrids must be standardized to enable regulatory approval.

Importantly, advancements in synthetic biology and nanotechnology are rapidly creating opportunities to engineer bacteria with enhanced safety profiles and design nanoparticles with improved efficiency in energy conversion and light penetration. Prioritizing biosafety and immune interactions as the immediate research focus, followed by addressing technical challenges of light delivery and scalable manufacturing, will provide the clearest path toward clinical adoption. By bridging these interdisciplinary innovations, bacteria–nanoparticle systems could transition from proof-of-concept studies to clinically viable theranostic platforms. Despite the remaining hurdles of immunogenicity, long-term biosafety, and large-scale production, this strategy highlights a transformative path for enhancing RT and improving cancer treatment outcomes in the future.

## Figures and Tables

**Table 1:** Anaerobic bacterial-nanoparticle strategies for hypoxia modulation in radiosensitization: a comprehensive review.

Year [refs]	Radiosensitizing effect	Complementary techniques	Hypoxia mitigation method	Cells and tumor models	In vitro/in vivo/injection mode	Radiation type	Radiation dose	Concentration of nanomaterials delivered	Functionalization/coating	Hydrodynamic size/shape	Surface charge/synthesis method	Nanoparticle name (commercial or by the authors)	Bacterial strain
2023 [24]	↑ROS production and induces DNA damage (formation of γ-H2AX foci)	PTT	Generating ROS	CT26 (colon carcinoma)	In vitro/in vivo (BALB/C mice)/intravenous	X-ray and 808 nm laser	0–2 Gy	50 µg/mL	DSPE-PEG2000-NH_2_ and DSPE-PEG2000-Cy5	BPQD (size: 3 nm, thickness: 1–2 nm), *E. coli* (size: 2.3 µm)	−11.2 mV/liquid exfoliation	Black phosphorus quantum dot (BPQD)	*Escherichia coli* (*E. coli*)

2022 [29]	↑ClyA secretion, shifting cell cycle S → G2/M	NR	The Bac@BNP system ↑generating ROS	4T1 breast cancer	In vitro/in vivo (BALB/C mice)/NR	X-rays	0–10 Gy	25 μg/mL of BNP and 1 × 10^8^ CFU/mL of bacteria	PLGVR peptide-modified BNPs	100 nm/spherical	−39.4 mV/amide condensation reaction	Bi_2_S_3_ nanoparticles (BNPs)	*Escherichia coli* MG1655

2025 [31]	↑Lactate consumption, oxygen generation, and inhibition of hypoxia-inducible factors	NR	SO@Hf-MOF-Pt biohybrid catalyzes H_2_O_2_ into oxygen, lactate consumption, and ROS production	CT26 colorectal cancer	In vitro/in vivo (BALB/C mice)/intravenous	X-ray	0–6 Gy	0.4 mg NPs and 5 × 10^7^ CFU bacteria	Hf-MOF-Pt NPs functionalized with Cy5-NHS and coated with SO	150 nm/nanocrystalline	+22 mV/in situ growth	Hf-MOF-Pt	*Shewanella oneidensis* MR-1 (SO)

2020 [32]	NSs generate ROS via FRET to RB, enhancing tumor cell death. *Clostridium novyi*-NT spores degrade tumor cells in hypoxic regions	PDT, MRI	Generating ROS via FRET	PC3 cells (prostate cancer)	In vitro/in vivo (BALB/C)/intratumoral	X-ray	0–6 Gy	60 μg/mL	Photosensitizer (rose bengal, RB) is coated on PS-NSs for FRET	40.03 nm/spherical	+38.3 mV/thermal decomposition method	PS-NSs (synthesized as NaGdF4: Tb, Ce@NaGdF4 core/shell)	*Clostridium novyi*-NT

*Note:* Hf-MOF-Pt, hafnium metal-organic framework-functionalized platinum nanoparticles.

Abbreviations: CFU, colony-forming units; FRET, fluorescence resonance energy transfer; MRI, magnetic resonance imaging; NCs, nanoclusters; NIR-II, near-infrared-II; NR, not reported; NSCLC, non-small cell lung cancer; PDT, photodynamic therapy; PLL, polylysine; PMC, photosynthesis microcapsule; PS-NSs, photosensitizer-coated nanoscintillator; PTT, photothermal therapy; UCNPs, upconversion nanoparticles.

**Table 2:** Cyanobacterial-nanoparticle strategies for hypoxia modulation in radiosensitization: a comprehensive review.

Year [refs]	Radiosensitizing effect	Complementary techniques	Hypoxia mitigation method	Cells and tumor models	In vitro/in vivo/injection mode	Radiation type	Radiation dose	Concentration of nanomaterials delivered	Functionalization/coating	Hydrodynamic size/shape	Surface charge/synthesis method	Nanoparticle name (commercial or by the authors)	Bacterial strain
2024 [33]	Generating ROS (particularly O_2_^•−^) and radiosensitizing properties of Au NCs	PDT	Oxygen production via photosynthesis and catalytic activity, converting oxygen into O_2_^•−^	4T1 and A549 breast and NSCLC cancer	In vitro/in vivo (BALB/C mice)/intravenous and intratumoral injection	X-ray and red-light illumination	0–9 Gy	500 μg/mL	Carboxymethyl chitosan-coated Au NCs	Helical structures of SP with granular Au NCs	NR/ionic crosslinking of Ca^2+^	Au NCs	Spirulina platensis (SP)

2022 [23]	Improving oxygenation	PDT	Photosynthesis and generating ROS	LLC-lung xenograft (C57/B6) and 4T1-breast xenograft (BALB/C)	In vitro/in vivo (BALB/C and C57/B6)	X-ray and 660 nm laser	0–6 Gy	200 μg/mL	Bismuthene nanosheets functionalized with oxygen	356 nm/nanosheets	−1.57 mV/freeze–thaw cycles and sonication	Bismuthene nanosheets (2D bismuthene)	*Synechococcus elongatus* PCC7942

2022 [27]	Hyperoxia induced by NIR–PMCs and synergistic lipid peroxidation	PDT	Oxygen production via evolved cyanobacteria encapsulated in PMCs	B16 melanoma cells	In vitro/in vivo (C57BL/6)	X-rays and NIR-II	300 mW/cm^2^ for NIR-II (45 min), 0–6 Gy for X-rays	0.7 µg UCNPs and 1 × 10^6^ cyanobacteria cells per PMC	Coated with PLL to prevent leakage	~20 nm/spherical	NR	UCNPs (specifically NaErF_4_: Tm@NaYF_4_)	Synechococcus elongates 7942

2020 [30]	↑DNA damage and ROS production	PDT, magnetic guidance and control	Oxygen generation in situ by MSP	SKOV3 (ovarian cancer), 4T1 (breast cancer), CT26 (colon cancer), HEK293 (kidney cells), HepL (hepatocytes)	In vitro/in vivo (BALB/C)/intravenous	X-ray and 650 nm laser	0–8 Gy	MSP with varying Fe_3_O_4_ NPs concentrations (0, 1.25, and 2.5 equivalent concentrations)	Surface modified with positively charged Fe_3_O_4_ NPs	MSP-2.5 (magnetic *S. platensis*): 44 nm, Fe_3_O_4_ NPs: ~10 nm	Fe_3_O_4_ coating (due to positive charge) deposition on *S. platensis*	Magnetic Fe_3_O_4_ NPs	Spirulina platensis

## Data Availability

This article is a review and does not contain any new data generated or analyzed by the authors.
